# Cross-editing by a tRNA synthetase allows vertebrates to abundantly express mischargeable tRNA without causing mistranslation

**DOI:** 10.1093/nar/gkaa469

**Published:** 2020-06-02

**Authors:** Meirong Chen, Bernhard Kuhle, Jolene Diedrich, Ze Liu, James J Moresco, John R Yates III, Tao Pan, Xiang-Lei Yang

**Affiliations:** Department of Molecular Medicine, Scripps Research Institute, La Jolla, CA 92037, USA; College of Food Science and Technology, Nanjing Agricultural University, Nanjing 210095, China; Department of Molecular Medicine, Scripps Research Institute, La Jolla, CA 92037, USA; Department of Molecular Medicine, Scripps Research Institute, La Jolla, CA 92037, USA; Department of Molecular Medicine, Scripps Research Institute, La Jolla, CA 92037, USA; Department of Molecular Medicine, Scripps Research Institute, La Jolla, CA 92037, USA; Department of Molecular Medicine, Scripps Research Institute, La Jolla, CA 92037, USA; Department of Biochemistry and Molecular Biology, The University of Chicago, Chicago, IL 60637, USA; Department of Molecular Medicine, Scripps Research Institute, La Jolla, CA 92037, USA

## Abstract

The accuracy in pairing tRNAs with correct amino acids by aminoacyl-tRNA synthetases (aaRSs) dictates the fidelity of translation. To ensure fidelity, multiple aaRSs developed editing functions that remove a wrong amino acid from tRNA before it reaches the ribosome. However, no specific mechanism within an aaRS is known to handle the scenario where a cognate amino acid is mischarged onto a wrong tRNA, as exemplified by AlaRS mischarging alanine to G4:U69-containing tRNA^Thr^. Here, we report that the mischargeable G4:U69-containing tRNA^Thr^ are strictly conserved in vertebrates and are ubiquitously and abundantly expressed in mammalian cells and tissues. Although these tRNAs are efficiently mischarged, no corresponding Thr-to-Ala mistranslation is detectable. Mistranslation is prevented by a robust proofreading activity of ThrRS towards Ala-tRNA^Thr^. Therefore, while wrong amino acids are corrected within an aaRS, a wrong tRNA is handled *in trans* by an aaRS cognate to the mischarged tRNA species. Interestingly, although Ala-tRNA^Thr^ mischarging is not known to occur in bacteria, *Escherichia coli* ThrRS also possesses robust cross-editing ability. We propose that the cross-editing activity of ThrRS is evolutionarily conserved and that this intrinsic activity allows G4:U69-containing tRNA^Thr^ to emerge and be preserved in vertebrates to have alternative functions without compromising translational fidelity.

## INTRODUCTION

Aminoacyl-tRNA synthetases (aaRSs) establish the rules for genetic code expression by matching each of the 20 proteinogenic amino acids to their cognate transfer RNAs (tRNAs), which harbor anticodon trinucleotides to allow the ‘translation’ of mRNA into proteins within the ribosome (1). Faithful translation of the genetic information is of central importance in biology (2). Because the accuracy of the aaRSs in pairing tRNAs with their cognate amino acids is greater than that of subsequent steps of ribosomal protein synthesis (3), the fidelity of translation is predominately dictated by aaRSs.

The aaRS-catalyzed tRNA aminoacylation is a two-step reaction: first, the amino acid is activated with ATP to form an enzyme-bound aminoacyl-adenylate; second, the aminoacyl moiety of the adenylate is transferred onto its cognate tRNA to generate the aminoacyl-tRNA product (4). To ensure the accuracy in aminoacylation of tRNAs, elaborate mechanisms of recognition for both the correct amino acid and the cognate tRNA by an aaRS have been evolved. The amino acid binding pocket at the active site of an aaRS plays the major role in identifying the correct amino acid. However, for certain aaRSs, the active site is not sufficient in selecting out the cognate amino acid due to high similarity with some noncognate amino acids in size and/or chemical properties. For example, serine can be misactivated by both AlaRS and ThrRS (5,6). Therefore, an editing domain has been incorporated into each synthetase to selectively hydrolyze the noncognate aminoacyl-adenylate (pre-transfer editing) or remove the noncognate amino acid from tRNA (post-transfer editing) (7–9). The importance of editing has been extensively demonstrated, as even mild editing defects will cause severe diseases (10).

As for the cognate tRNA recognition, it often involves the anticodon and the acceptor stem of the tRNA to be specifically identified by the anticodon binding domain and the catalytic domain, respectively, of the corresponding aaRS. Mischarging a cognate amino acid onto a noncognate tRNA is less frequently reported (11–15). In this scenario, because the amino acid is cognate to the synthetase, neither pre- nor post-transfer editing is effective to remove the error. A recent study found that, under stress conditions, MetRS could misacylate methionine onto various noncognate tRNAs. The lack of editing of the mischarged noncognate tRNAs leads to mis-incorporation of methionine into proteins, which could protect cells against oxidative damage (11). Although mistranslation may provide beneficial effects for a short term as in this case, long-lasting mistranslation is likely to be detrimental for cells.

Interestingly, certain aaRSs are prone to mischarging of noncognate tRNAs. For example, AlaRS, which lacks an anticodon binding domain, recognizes its cognate tRNA based on a single G3:U70 base pair in the acceptor stem (16), and thus is prone to potential perturbation in pairing accuracy (14,17). Indeed, using a tRNA microarray system, we detected that human AlaRS can mischarge alanine onto noncognate tRNAs with a G4:U69 base pair, including tRNA^Cys^ and tRNA^Thr^ (14). Although AlaRS can mischarge both tRNA^Cys^ and tRNA^Thr^, we only detected a cysteine-to-alanine, but not threonine-to-alanine, substitution in a reporter protein expressed in human cells (14), suggesting the existence of a trans-editing mechanism to specifically remove the mischarged alanine from tRNA^Thr^ but not tRNA^Cys^, among other possible explanations.

In this work, we extensively studied the mischargeable G4:U69-containing tRNA^Thr^ to understand its apparent lack of mistranslation in human cells. We found that the mischargeable tRNA^Thr^ species are ubiquitously and highly expressed among various mammalian cell lines and tissues. Upon rigorous analysis, we again failed to detect the corresponding Thr-to-Ala mistranslation in the human proteome. We identified a robust cross-editing mechanism that removes the mischarged alanine from tRNA^Thr^. While AlaRS itself is unable to correct this mistake, ThrRS efficiently deacylates the mischarged Ala-tRNA^Thr^ at its editing site. Therefore, while wrong amino acids are corrected within an aaRS, a wrong tRNA is handled *in trans* by an aaRS cognate to the mischarged tRNA species. AlaRS and ThrRS thus constitute a mischarging-editing cycle which protects the cell from noncognate tRNA charging and its detrimental effects. We outline a process by which organisms can evolve novel translation-independent functions of specialized tRNA species without compromising translational fidelity.

## MATERIALS AND METHODS

### Antibodies for western blot

Antibodies used in this research include mouse anti-V5 (R96-CUS, Invitrogen, Grand Island, NY, USA), rabbit anti-β-actin (13E5, Cell Signal Technology, Danvers, MA, USA), rabbit anti-human ThrRS (A304-755A, Bethyl Laboratories, Montgomery, TX, USA), and rabbit anti-human ATD (C14orf126-Antibody-C-term, Abgent, San Diego, CA, USA).

### *In vitro* transcription of tRNA

Human tRNA^Thr^ and tRNA^Ala^ was *in vitro* transcribed as described previously with modification (18). Template DNA for tRNA transcription was amplified by PCR. After 5-h transcription reaction at 37°C, the reaction was terminated and the product loaded onto a DEAE column (GE healthcare, USA) equilibrated in low salt buffer containing 50 mM MES pH 6.5, 150 mM NaCl, 0.2 mM EDTA to remove proteins and NTPs. The tRNA was eluted with high salt buffer containing 400 mM NaCl. tRNA fractions were pooled and precipitated by ethanol. The purity of tRNA was checked by urea-PAGE. Prior to use in assays, tRNA was incubated at 80°C for 5 min followed by refolding at 60°C in 5 mM MgCl_2_ and gradually cooling down to room temperature.

### Northern blot

RNA samples, either transcribed tRNAs or total RNA from HEK293 cells and mouse tissues, were separated by 15% urea–PAGE, followed by electrophoretic transfer onto a nylon membrane. The crosslinked membrane was hybridized with DNA probes ([5′-^32^P] labeled using T4 PNK) at a specific temperature for 20 h. The washed membrane was incubated with phosphor imaging plate to develop blots. For detection of different RNAs, the membrane was stripped of the previous probe each time before the next one was applied. After stripping, the absence of residual activity from the previous probe was checked on a phosphorimager. A pre-stained marker for small RNA series was added in a separate lane and was cut from the membrane before hybridization. The two membrane fragments were subsequently aligned to indicate the size of RNAs. *In vitro* transcribed and purified tRNAs were used to precisely indicate the size of the tRNAs purified from cells and tissues.

### Design and testing of DNA probes for northern blot analysis

The oligomer sequences of probes for northern blot were designed to specifically recognize the two tRNA^Thr^(G4:U69)-AGU isodecoders (Thr-AGU-4 and Thr-AGU-7), both tRNA^Thr^(G4:U69)-CGU isodecoders (Thr-CGU-3 and Thr-CGU-5), or non-G4:U69-containing tRNA^Thr^ (Thr-AGU-6) (Supplementary Figures S3 and S4A). (Thr-AGU-6 was selected as control as it has the highest sequence similarity to all four tRNA^Thr^(G4:U69) genes, making it the most stringent control to probe specificity.) Specificity and sensitivity of all probes were examined against purified tRNA transcripts, using the northern blot protocol outlined above. The probe for tRNA^Thr^(G4:U69)-CGU (pbThr-CGU(G4:U69)) recognizes transcribed Thr-CGU-5 in a concentration-dependent manner but does not recognize the control Thr-AGU-6 (Supplementary Figure S4B). Similarly, the probe for tRNA^Thr^(G4:U69)-AGU (pbThr-AGU(G4:U69)) recognizes Thr-AGU-7 in a concentration-dependent manner but also weakly recognizes the control Thr-AGU-6, which differs from Thr-AGU-4/7 by only one nucleotide in the region covered by the probe, at its highest concentration (Supplementary Figure S4C). The probe for the control tRNA (pbThr-AGU-6) is highly specific and did not recognize Thr-AGU-7 even at its highest concentration (Supplementary Figure S4C). Importantly, both probes showed high specificity in cell-based detection assays, where Thr-AGU-7 overexpressed in HEK293 cells could be detected only by pbThr-AGU(G4:U69) but not by pbThr-AGU-6 and vice versa (Supplementary Figure S4C). 5S rRNA was used as an internal reference, using the following oligomer probe: 5′-CATCCAAGTACTAACCAGGCCCGAC-3′.

### Expression and purification of recombinant proteins from *E. coli*

Coding sequences for AlaRS and ThrRS were cloned into plasmid pET-21a, respectively. ThrRS mutants (H155A/H159A, D259A) were constructed by the Quickchange method. The recombinant proteins with his-tag were first purified by affinity chromatography, and then the eluted proteins went through ion exchange column, HiTrap Heparin (GE Healthcare) for ThrRS and its mutants while HiTrap Q for AlaRS. Finally, proteins were loaded onto HiLoad 200 16/60 and eluted proteins were pooled and concentrated. Proteins at each step of purification were monitored by SDS-PAGE.

### Protein and tRNA overexpression in mammalian cells

Genes encoding human AlaRS, GAPDH and tRNAs were cloned into the pCDNA6-V5/His-C vector (Life Technologies, Grand Island, NY, USA), respectively. The plasmids were transfected into HEK293 cells using Lipofectamine 3000 (Life Technologies, Grand Island, NY, USA), which were further cultured for 48 h in DMEM supplemented with 10% fetal bovine serum (FBS). The cells were harvested and washed with cold PBS and stored for further use.

### Aminoacylation and deacylation assays

The aminoacylation assays were performed as described previously (14). The reactions were incubated with 50 mM HEPES pH 7.5, 20 mM KCl, 5 mM MgCl_2_, 4 mM ATP, 4 mM DTT, 4 μg/ml pyrophosphatase, 40 μM cold l-alanine, 3.58 μM [^3^H]Alanine (1mCi/ml) and 20 μM tRNA^Thr^, or 4 μM tRNA^Ala^. The reactions were initiated by adding 1 μM AlaRS or ThrRS (Figure 2A). The comparison of the charging activity of AlaRS toward different cognate and noncognate tRNAs was performed under conditions using 200 nM AlaRS and 4 μM tRNA^Thr^(G3:U70) or tRNA^Ala^, or 20 μM tRNA^Thr^(G4:U69) (Figure 2B). For mischarging by ThrRS or its mutant ThrRS^H155A/H159A^, the reaction was carried out with 200 nM enzyme and 4 μM tRNA^Thr^ (Figure 3D). For alanylation of tRNA^Thr^(G4:U69) by AlaRS in the presence of ThrRS, the reaction was carried out with 1 μM AlaRS and 20 μM tRNA^Thr^(G4:U69), with or without 500 nM ThrRS/ThrRS^H155A/H159A^ (Figure 3E). At varying time intervals, 5 μl aliquots were applied to MultiScreen 96-well filter plate pre-wetted with quench solution containing 0.5 mg/ml DNA and 100 mM EDTA in 300 mM NaOAc (pH 3.0), followed by the same procedures described in previous paper (14). For deacylation assay, 2 μM [^3^H]Ala-tRNA^Thr^ was incubated with 200 nM ThrRS, ThrRS mutants, or ATD in the buffer containing 50 mM HEPES pH 7.5, 20 mM KCl, 5 mM MgCl_2_, 0.2 mg/ml BSA. Reactions were quenched at various time points, and the deacylation rate was calculated based on the reduced [^3^H]Ala-tRNA^Thr^ signals. All raw data from the aminoacylation and deacylation assays are included as Supplementary Table S5.

### Electrophoretic mobility shift assay

Each tRNA (400 nM) was incubated with increasing amount of AlaRS for 30 min at room temperature. The tRNA-protein complex was resolved on a Native PAGE containing 10% acrylamide in Tris-glycine buffer.

### Identification of Thr-to-Ala misincorporation by mass spectrometry

For mass spectrometry (MS) analysis of HEK293 cell lysate, the proteins were extracted by methanol/chloroform, which were digested by trypsin and further desalted before loading onto MS. For MS analysis of recombinant GAPDH, GAPDH was isolated and enriched by immunoprecipitation using mouse V5 antibody, which was digested and desalted before MS analysis. For MS analysis of luciferase protein, luciferase gene with an amber stop codon at Thr348 position was inserted into pCDNA6 vector, which was co-transformed into HEK293 with pCDNA6 vector containing tRNA^Thr^(G4:U69) gene or the chimeric positive control tRNA^Thr^(G3:U70) with the anticodon changed into that corresponds to amber stop codon. The full-length luciferase protein with the read-through of amber stop codon was enriched by immunoprecipitation using mouse V5 antibody, and applied for MS analysis as above.

The digested samples were analyzed on a Q Exactive HFX mass spectrometer (Thermo). Samples were injected directly onto a 25 cm, 100 μm ID column packed with BEH 1.7 μm C18 resin (Waters). Samples were separated at a flow rate of 300 nl/min on a nLC 1200 (Thermo). Buffer A and B were 0.1% formic acid in water and 90% acetonitrile, respectively. A gradient of 1–25% B over 180 min, an increase to 40% B over 40 min, an increase to 90% B over another 10 min and held at 90% B for a 10 min was used for a 240 min total run time. Column was re-equilibrated with 15 μl of buffer A prior to the injection of sample. Peptides were eluted directly from the tip of the column and nanosprayed directly into the mass spectrometer by application of 2.8 kV voltage at the back of the column. The Q Exactive was operated in a data dependent mode. Full MS scans were collected in the Orbitrap at 120K resolution with a mass range of 400–2000 *m*/*z*. The 10 most abundant ions per cycle were selected for MS/MS and dynamic exclusion was used with exclusion duration of 10 sec.

Protein and peptide identification were done with Integrated Proteomics Pipeline – IP2 (Integrated Proteomics Applications). Tandem mass spectra were extracted from raw files using RawConverter (19) and searched with ProLuCID (20) against human UniProt database. The database was appended with the sequence of Luciferase where each of the 20 amino acids are possible at the amber stop codon site. The search space included all fully-tryptic and half-tryptic peptide candidates. Carbamidomethylation on cysteine was considered as a static modification. Data was searched with 50 ppm precursor ion tolerance and 600 ppm fragment ion tolerance. Identified proteins were filtered using DTASelect (21) and utilizing a target-decoy database search strategy to control the false discovery rate to 5% at the spectrum level. The spectra covering the amber codon stop site were manually validated (22).

## RESULTS

### G4:U69-containing mischargeable tRNA^Thr^ are ubiquitously and abundantly detected in mammalian cells and tissues

**Figure 1.**
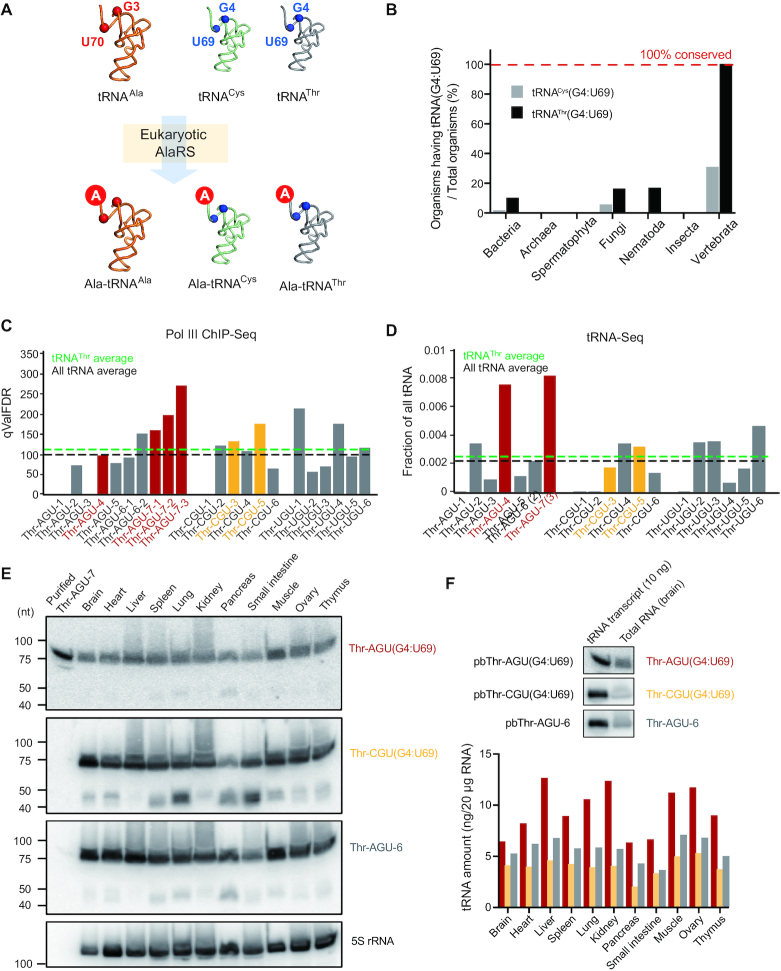
Conservation and expression of G4:U69-containing tRNA^Thr^ in vertebrates. (**A**) Illustration that tRNA^Cys^ and tRNA^Thr^ that contain a G4:U69 wobble base pair can be mischarged with alanine by eukaryotic AlaRS. (**B**) Conservation analysis of G4:U69-containing tRNA^Cys^ and tRNA^Thr^ in different organisms based on the tRNA database (http://gtrnadb.ucsc.edu). (**C**, **D**) Pol III ChIP-Seq (C) and tRNA-Seq (D) results of tRNA^Thr^ genes in HEK293 or HEK293T cells, based on published data from Oler A.J. *et al.* (2010) and Zheng, G. *et al.* (2015), respectively. G4:U69-containing tRNA^Thr^ belonging to AGU and CGU isoacceptors are marked in red and orange, respectively. The black and green dash line indicates the average level of all tRNA and all tRNA^Thr^, respectively. (**E**) Northern blot analysis of tRNA^Thr^ genes in mouse tissues. For each sample, 20 μg of total RNA was loaded and 5S rRNA was used as an internal reference. (**F**) Quantification of the expression of tRNA^Thr^ genes as detected in (**E**) using *in vitro* transcribed tRNAs (10 ng) as standards.

According to our previous tRNA microarray analysis, both human cytoplasmic tRNA^Cys^ and tRNA^Thr^ can be mischarged with alanine by AlaRS (Figure 1A), with significantly higher levels of mischarged Ala-tRNA^Thr^ than for Ala-tRNA^Cys^(14). Analysis of the tRNA genome showed that the number of G4:U69-containing tRNA^Thr^ genes (hereafter denoted as tRNA^Thr^(G4:U69)) is greater than that of G4:U69-containing tRNA^Cys^ genes (hereafter tRNA^Cys^(G4:U69)) in human and mouse cells (Supplementary Tables S1–S4). Moreover, in vertebrates including mammals, tRNA^Thr^(G4:U69) genes, but not tRNA^Cys^(G4:U69) genes, are absolutely conserved in all genetically annotated organisms (Figure 1B), suggesting the existence of strong selective pressure to retain the G4:U69 wobble base pair within tRNA^Thr^ genes in vertebrates.

**Figure 2.**
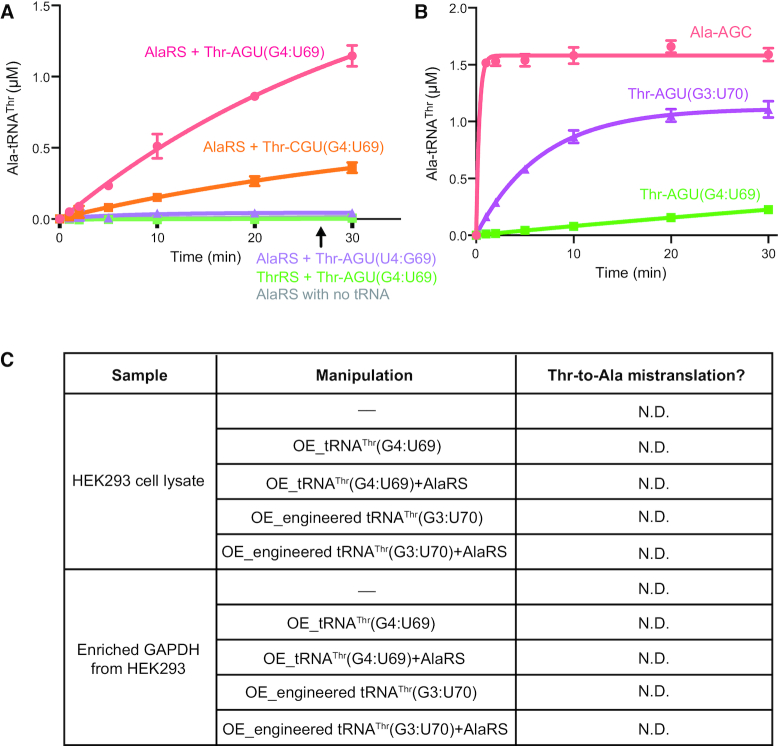
Lack of Thr-to-Ala mistranslation despite effective mischarging of Ala to tRNA^Thr^(G4:U69). (**A**) Both Thr-AGU(G4:U69) and Thr-CGU(G4:U69), but not Thr-AGU(U4:G69), can be mischarged with Ala by AlaRS but not ThrRS. The reaction was carried out with 1 μM AlaRS or ThrRS and 20 μM tRNA^Thr^; AlaRS alone without tRNA was used as negative control. (**B**) Chimeric tRNA^Thr^ with G3:U70 (made by switching the original C3:G70 and G4:U69 base pairs) can be mischarged with Ala by AlaRS with enhanced efficiency. The reaction was carried out with 200 nM AlaRS and 4 μM Ala-AGC or Thr-AGU(G3:U70) tRNA, or 20 μM Thr-AGU(G4:U69) tRNA. (**C**) No Thr-to-Ala substitution was observed in either HEK293 cell lysate or enriched GAPDH from HEK293 cells even under various indicated manipulations to enhance Ala mischarging. OE: overexpression; N.D.: not detected.

Among the 19 different tRNA^Thr^ isoacceptors and isodecoders (encoded by 22 genes) in human cells, 4 possess a G4:U69 base pair (encoded by six genes), with two each in the AGU and CGU isoacceptor families (Supplementary Figure S1, Table S1). A similar situation is found in mouse cells (Supplementary Table S2). To gain insight whether either isoacceptor can be mischarged with alanine, we tested two human transcripts – Thr-AGU-7(G4:U69) and Thr-CGU-5(G4:U69) (annotated as Thr-AGU(G4:U69) and Thr-CGU(G4:U69), respectively, for simplicity). Because most non-G4:U69-containing tRNA^Thr^ isoacceptors have U4:G69 (Supplementary Table S1), we mutated the G4:U69 base pair in Thr-AGU(G4:U69) to U4:G69 (annotated as Thr-AGU(U4:G69)) and used it as a negative control. Using an *in vitro* aminoacylation assay, we confirmed that both tRNA^Thr^(G4:U69) isoacceptor transcripts were efficiently mischarged by AlaRS, while no mischarging was observed with tRNA^Thr^(U4:G69) (Figure 2A). The stronger mischarging of Thr-AGU(G4:U69) compared to Thr-CGU(G4:U69) isoacceptors suggests that sequence elements other than G4:U69 may also contribute to mischarging (Figure 2A).

The effect of mischarging by AlaRS on translation would greatly depend on the expression levels of tRNA^Thr^(G4:U69) genes. To obtain insight into the expression of the six tRNA^Thr^(G4:U69) genes, we analyzed the Chromatin Immunoprecipitation (ChIP) data of RNA Polymerase III (Pol III) in human cells (23), and human and mouse liver tissues (24), where Pol III-binding indicates active transcription of tRNA genes. According to this analysis, tRNA^Thr^(G4:U69) genes show high Pol III occupancies in all four available cell lines—HEK293 (Figure 1C), HeLa, Jurkat, and HFF (Supplementary Figure S2A), as well as human and mouse liver tissues (Supplementary Figure S2B). Most of the tRNA^Thr^(G4:U69) genes showed higher Pol III binding than the average for all tRNA^Thr^ genes (Figure 1C and Supplementary Figures S2A, B), suggesting relatively high transcription levels of tRNA^Thr^(G4:U69) genes in these cell lines and liver tissues. Consistently, deep sequencing data for tRNA transcripts (tRNA-Seq) (25) directly show that tRNA^Thr^(G4:U69) isodecoders, in particular from the tRNA^Thr^(G4:U69)-AGU isoacceptor family, are the most abundant tRNA^Thr^ species expressed in HEK293T cells (Figure 1D and Supplementary Figure S2C). By contrast, low Pol III occupancy (Supplementary Figure S3A) and low tRNA transcript levels (Supplementary Figure S3B) were detected for the single tRNA^Cys^(G4:U69) gene in HEK293 cells. The latter may be related to its less stable secondary clover-leaf structure, as indicated by a low tRNAScan score (Supplementary Table S3).

To further confirm the expression of tRNA^Thr^(G4:U69) genes and to probe its potential tissue specificity, we used northern blot analysis to detect tRNA^Thr^(G4:U69) with either AGU or CGU anticodon in human cell lines and various mouse tissues. For this purpose, DNA probes were developed that specifically recognize both tRNA^Thr^(G4:U69)-AGU isodecoders (Thr-AGU-4 and Thr-AGU-7), both tRNA^Thr^(G4:U69)-CGU isodecoders (Thr-CGU-3 and Thr-CGU-5), or a control non-G4:U69-containing tRNA^Thr^ (Thr-AGU-6) (see Material and Methods and Supplementary Figure S4A). Using these probes, we were able to successfully detect abundant endogenous expression of tRNA^Thr^(G4:U69)-CGU, tRNA^Thr^(G4:U69)-AGU in HEK293 cells (Supplementary Figures S4B and S4C). Moreover, using the same probes, we found tRNA^Thr^(G4:U69) from AGU and CGU isoacceptor families to be ubiquitously expressed across different mouse tissues (Figure 1E and Supplementary Figure S4D). Quantification analysis against purified tRNA transcripts indicates higher levels of tRNA^Thr^(G4:U69)-AGU compared to tRNA^Thr^(G4:U69)-CGU in all tissue types (Figure 1F), consistent with the tRNA-Seq data from HEK293 cells (Figure 1D). Overall, combining Pol III-ChIP, tRNA-Seq and northern blot analyses, we demonstrate that tRNA^Thr^(G4:U69) genes are ubiquitously and abundantly expressed in the mammalian system.

### Mischarging of alanine onto tRNA^Thr^(G4:U69) does not yield mistranslation

The conservation and abundant expression of the mischargeable tRNA^Thr^(G4:U69) species pose the question whether it can lead to mistranslation. Although beneficial effects of deliberate modifications of translation fidelity have been documented (26), widespread mistranslation would be detrimental. In order to investigate the tRNA^Thr^(G4:U69)-mediated mistranslation, we exhaustively explored this possibility in HEK293 cells by mass spectrometry analysis as detailed below.

To generate a positive control, we created the chimeric tRNA^Thr^(G3:U70) by moving up the GU base pair in a natural tRNA^Thr^(G4:U69) (i.e. Thr-AGU-7) along the acceptor stem of the tRNA (Supplementary Figure S5) to enhance its capacity to be mischarged by AlaRS (Figure 2B). To ensure that we can detect potential threonine-to-alanine substitution by mass spectrometry analysis, we incorporated a translation readthrough strategy (Supplementary Figure S6A). The gene of the reporter protein (luciferase) contains an amber stop codon TAG in the middle and the DNA sequence encoding a V5-tag at the C-terminus. Only when the stop codon is readthrough by tRNAs with an CUA anticodon, the full-length protein is expressed with a V5-tag for capture. We replaced the anticodon to CUA in the chimeric tRNA^Thr^(G3:U70) and the natural tRNA^Thr^(G4:U69) to allow the translation readthrough. The replacement should not affect mischarging by AlaRS because the AlaRS-tRNA recognition does not involve the anticodon (16). Indeed, when the amber tRNA^Thr^ (G3:U70) is expressed as the suppressor tRNA, out of the 21 readthrough peptides we detected, 19 contain an Ala, while only 2 have a Thr, at the amber stop codon site (Supplementary Figure S6B), suggesting that the amber tRNA^Thr^ (G3:U70) is mostly charged with alanine by AlaRS as expected and that threonine-to-alanine substitution in proteins can be successfully detected. In contrast, when the natural mischargeable tRNA^Thr^(G4:U69) with the engineered anticodon is expressed as the suppressor tRNA, out of the 14 readthrough peptides we detected, all of them had the cognate Thr at the amber stop codon position. Therefore, although the anticodon of tRNA^Thr^ is an identity element, tRNA^Thr^ containing the amber anticodon can still be recognized and aminoacylated by ThrRS, consistent with previous reports (27,28).

To further confirm the lack of Thr-to-Ala mistranslation in HEK293 cells, we used another reporter protein (GAPDH) without the readthrough design. In this case, we can directly overexpress the mischargeable tRNA^Thr^(G4:U69) with its natural anticodon. Intended as a positive control, the chimeric tRNA^Thr^(G3:U70) was separately overexpressed. However, no Thr-to-Ala substitution was found in the reporter protein with either tRNA overexpressed and when AlaRS co-overexpressed with each tRNA to further enhance the alanine mischarging (Figure 2C). We also attempted to detect a Thr-to-Ala substitution in the HEK293 whole cell lysate. Again, no mistranslation was detected even with the co-expression of chimeric tRNA^Thr^(G3:U70) and AlaRS (Figure 2C). Considering the sensitivity limitations of mass spectrometry, especially when the amount of the target peptide is low, we cannot rule out the possibility that mistranslation may happen below the detectable level. No obvious cellular toxicity was observed upon overexpression of the natural or the engineered tRNA^Thr^, consistent with the lack of detectable mistranslation.

### ThrRS is the main factor for editing Ala-tRNA^Thr^ to prevent mistranslation

The lack of detection of threonine-to-alanine substitutions in the reporter proteins as well as in the proteome of HEK293 cells suggests the existence of an editing activity that removes the mischarged alanine from tRNA^Thr^. Recently, a trans-editing factor ATD (Animalia-specific tRNA deacylase), also known as DTD2 (D-aminoacyl tRNA deacylase-2), was reported to deacylate Ala-tRNA^Thr^(G4:U69) (29). However, based on the HUMAN PROTEOME MAP database (http://www.humanproteomemap.org/) and the Human Protein Atlas (https://www.proteinatlas.org/), expression of ATD is barely detected in many tissues and cell types (Supplementary Figure S7A). Given the ubiquitous and abundant expression of tRNA^Thr^(G4:U69) (Figure 1E and F), it is therefore unclear whether ATD is sufficient or at all available for correcting Ala-tRNA^Thr^ in all tissues.

An alternative candidate for hydrolyzing the mischarged Ala-tRNA^Thr^ is ThrRS. ThrRS possesses an editing domain with known cis-acting post-transfer editing activity to remove noncognate amino acid from tRNA^Thr^ after it is being mischarged in the synthetic site (6). Importantly, ThrRS is ubiquitously and highly expressed in all cell and tissue types (Supplementary Figure S7A). We detected and quantified ATD and ThrRS levels in various cells including HEK293 cells. Indeed, the expression level of ThrRS is substantially higher than that of ATD in all cells (Supplementary Figures S7B and C). Particularly, in HEK293 cells, the concentration of ThrRS is about 5-fold higher than that of ATD (Supplementary Figures S7B and S7C).

**Figure 3.**
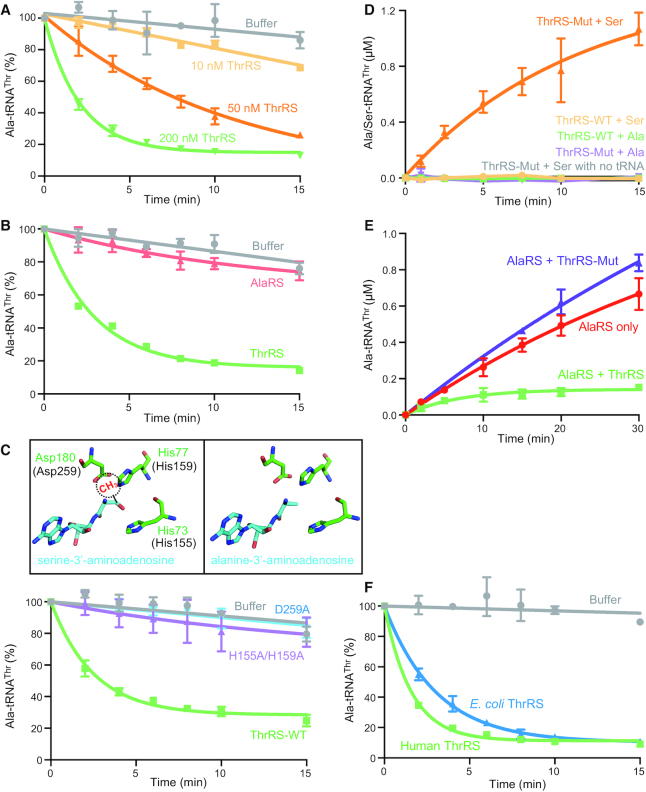
Cross-editing of Ala-tRNA^Thr^ by ThrRS. (**A**) Human ThrRS could efficiently deacylate Ala-tRNA^Thr^. Different concentrations of ThrRS as indicated were incubated with 2 μM ^3^H-labeled Ala-tRNA^Thr^. In the following assays, 200 nM enzyme (ThrRS or AlaRS) and 2 μM Ala-tRNA^Thr^ were used in the reaction unless specified. (**B**) AlaRS harboring an editing domain similar to that of ThrRS cannot deacylate Ala-tRNA^Thr^. (**C**) Residues in ThrRS involved in deacylation of Ser-tRNA^Thr^ are important for editing Ala-tRNA^Thr^. Top: alanine-3′-aminoadenosine, an analog of Ala-tRNA^Thr^ terminus, was modeled into editing site of *E. coli* ThrRS based on the structure of *E.coli* ThrRS in complex with serine-3′-aminoadenosine (PDB ID: 1TKY). The equivalent residue numbers in human ThrRS are shown in parenthesis. The methyl group of threonine is modeled and shown as a dashed circle, which would clash with Asp259 and His159. Bottom: Both D259A and H155A/H159A mutants completely abolish the editing activity of human ThrRS towards Ala-tRNA^Thr^. (**D**) Editing-defective ThrRS H155A/H159A mutant (ThrRS-Mut) can mischarge serine, but not alanine, onto tRNA^Thr^. (**E**) ThrRS can deacylate Ala-tRNA^Thr^ in the presence of AlaRS. ThrRS-Mut may slightly enhance the release of Ala-tRNA^Thr^ from AlaRS and thus the turnover of AlaRS. The reaction was carried out with 1 μM AlaRS and 20 μM tRNA^Thr^, with or without 500 nM ThrRS/ThrRS-Mut. (**F**) *E. coli* ThrRS is able to deacylate human Ala-tRNA^Thr^ with similar efficiency as human ThrRS.

Next, we investigated the potential deacylation activity of ThrRS against Ala-tRNA^Thr^ and compared it with that of ATD. Ala-tRNA^Thr^(G4:U69) can be rapidly hydrolyzed by ThrRS (Figure 3A), and the editing activity of ThrRS is more efficient than that of ATD (Supplementary Figure S8). Combining the activity and the expression analyses, ThrRS is more likely to be the main factor *in vivo* that edits Ala-tRNA^Thr^ to prevent mistranslation. Moreover, as expected, although AlaRS also have an editing domain, it cannot edit Ala-tRNA^Thr^ (Figure 3B).

### The editing domain active site of ThrRS is responsible for hydrolyzing Ala-tRNA^Thr^

Previous studies of the post-transfer editing activity of ThrRS focused on deacylating the mischarged Ser-tRNA^Thr^ (6). It was shown that, while the cognate threonine moiety is sterically excluded from the editing site, the absence of the methyl group in the side chain of serine allows it to fit into the editing domain active site pocket (Figure 3C) (30). This led us to assume that alanine, with an even smaller side chain, would fit into the editing pocket of ThrRS as well (Figure 3C). To confirm that hydrolysis of Ala-tRNA^Thr^ occurs in the editing site of ThrRS, we introduced editing site mutations, including a double mutant H155A/H159A and a single mutant D259A, (Figure 3C), each has been shown to impair editing of Ser-tRNA^Thr^ by ThrRS (6). Indeed, these mutations completely abolished the editing of Ala-tRNA^Thr^ (Figure 3C).

### ThrRS cannot mischarge alanine onto tRNA^Thr^ but can cross-edit the mischarged Ala-tRNA^Thr^

The erroneous capacity of ThrRS to activate serine and to generate Ser-tRNA^Thr^ necessitates the evolutionary conservation of its post-transfer editing activity (6,31). Our observation that ThrRS also edits Ala-tRNA^Thr^ raises the question whether ThrRS itself is able to mischarge alanine onto tRNA^Thr^. To address this point, firstly, we show that no Ala-tRNA^Thr^ is formed with ThrRS (Figure 2A). However, the lack of mischarging could result from the above demonstrated editing activity of ThrRS. Indeed, no Ser-tRNA^Thr^ can be detected with ThrRS either (Figure 3D). Yet, when the editing activity of ThrRS is abolished by the H155A/H159A mutation, only Ser-tRNA^Thr^, but not Ala-tRNA^Thr^, can be formed (Figure 3D), demonstrating that human ThrRS cannot mischarge alanine onto tRNA^Thr^ independent of its editing activity. Therefore, ThrRS only possesses cross-editing activity for Ala-tRNA^Thr^.

To further investigate the mischarging-editing-cycle of tRNA^Thr^(G4:U69) catalyzed by two different aaRSs, we monitored the mischarging of tRNA^Thr^(G4:U69) by AlaRS in the presence of ThrRS. In these experiments, although AlaRS was in excess of ThrRS (2-fold), the presence of ThrRS markedly reduced the accumulation of misacylated Ala-tRNA^Thr^ (Figure 3E). Importantly, this effect of ThrRS was abolished by the H155A/H159A mutation in the editing site. Moreover, the addition of ThrRS^H155A/H159A^ slightly enhanced the mischarging of tRNA^Thr^ (Figure 3E), presumably by competing with AlaRS for binding to Ala-tRNA^Thr^, thereby facilitating the turnover and the release of the mischarged tRNA from AlaRS.

### Prokaryotic ThrRS also possesses cross-editing ability to deacylate Ala-tRNA^Thr^

Our previous study found that eukaryotic, but not prokaryotic, AlaRS can mischarge tRNA with a G4:U69 base pair (14). The mischarging capacity is determined by several key residues in the tRNA binding domain of AlaRS, which are divergent between eukaryotes and prokaryotes (14). Interestingly, although the mischarged Ala-tRNA^Thr^ is unlikely to exist in prokaryotes, *E. coli* ThrRS, like human ThrRS, can cross-edit the mischarged Ala-tRNA^Thr^(G4:U69) with an efficiency similar to that of the human enzyme (Figure 3F). Therefore, ThrRS seems to have an inherent capacity to edit Ala-tRNA^Thr^, regardless of the existence of the mischarged species within the biological system.

## DISCUSSION

Aminoacylation is a strictly controlled process with multiple proofreading mechanisms ensuring high accuracy. While numerous mechanisms have been described for how aaRSs prevent misacylation of noncognate amino acids onto their cognate tRNA, no editing mechanisms have been described so far for cases in which an aaRS mischarges its cognate amino acid onto a noncognate tRNA. In fact, MetRS could mischarge methionine onto various noncognate tRNAs, which would then, due to the absence of any proofreading mechanism to clear the mistake, be used in translation (11,12). Using a microarray assay, we previously found that alanylation of tRNA^Thr^ accounts for 58% of all misalanylated tRNA. In contrast, tRNA^Cys^ accounts for only 5%, and yet misincorporation of alanine in lieu of cysteine could be detected by mass spectrometry. This suggests that the levels of Ala-tRNA^Thr^ should be high enough to cause mistranslation if it was not cleared by editing factors (14). However, we show here that mischarging of tRNA^Thr^ by AlaRS unlikely results in mistranslation presumably due to the novel cross-editing activity of ThrRS. Although other factors such as ATD may contribute to removing mischarged alanine from tRNA^Thr^, the higher *in vitro* activity and its ubiquitous expression as a housekeeping protein suggest that ThrRS is the main factor responsible for editing Ala-tRNA^Thr^*in vivo*.

It would be ideal if we can demonstrate the significance of the cross-editing activity of ThrRS *in vivo*. In theory this may be achieved by creating cell lines that are defective in ThrRS editing to reveal the otherwise corrected alanine mischarging of tRNA^Thr^ and Thr-to-Ala mistranslation. However, we have shown that the same editing site used to correct the mischarged alanine-tRNA^Thr^*in trans* is also used to correct commonly mischarged serine-tRNA^Thr^*in cis*, indicating that the editing activity of ThrRS is likely to be indispensable for cell viability. Indeed, it has been demonstrated that a similar editing activity from AlaRS is essential. Mouse homozygous in expressing a severe editing-deficient AlaRS (AlaRS-C723A) died at early stage of embryonic development (32). It is worth noting that a ThrRS-like protein (TARSL2) has been identified in higher eukaryotes and was demonstrated to have both tRNA aminoacylation and editing activities (33). The editing domain of TARSL2 is highly homologues to that of the canonical ThrRS, including strict conservations of the key residues for editing, indicating TARSL2 would also be capable to trans-edit the mischarged alanine-tRNA^Thr^. The existence of both ThrRS and a ThrRS-like protein would further explain why vertebrates can abundantly express mischargeable tRNA^Thr^ without causing mistranslation.

**Figure 4.**
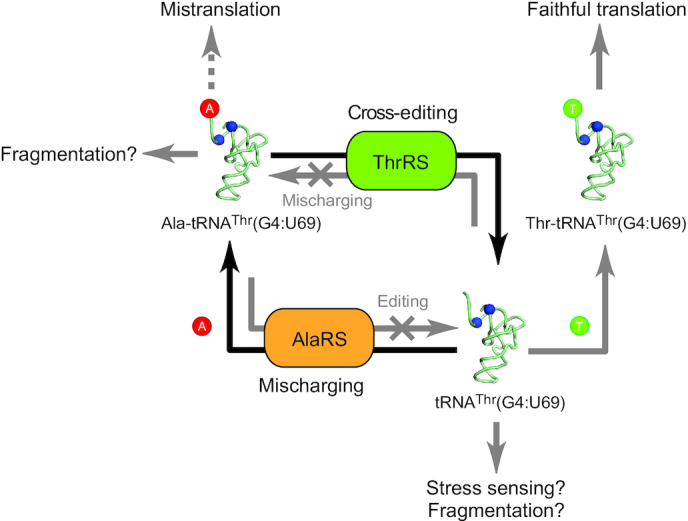
The proposed mechanism through the cycle of AlaRS-mediated mischarging and ThrRS-mediated cross-editing of tRNA^Thr^(G4:U69) to allow vertebrates to express the mischargeable tRNA^Thr^(G4:U69) without causing mistranslation and the speculated biological functions of the mischargeable tRNA. In vertebrates, not only can tRNA^Thr^(G4:U69) be correctly charged with Thr by ThrRS, it can also be mischarged with Ala by AlaRS to some extent, while AlaRS with editing domain cannot hydrolyze the mischarged Ala-tRNA^Thr^. However, the mistake can be corrected by ThrRS through a cross-editing mechanism, whereas ThrRS itself is unable to mischarge Ala onto tRNA^Thr^. The cross-editing function of ThrRS can prevent deleterious effects caused by mistranslation in organisms, and thus allow the conservation of mischargeable tRNA^Thr^, probably for alternative functions beyond translation, such as stress sensing or other regulatory functions through tRNA fragmentations.

Editing of a cognate amino acid from a noncognate tRNA poses a unique challenge to synthetases. Given that the correct amino acid is being used, neither classical pre- nor post-transfer editing mechanisms would recognize the mistake. In addition, most editing mechanisms employed by aaRSs also rely on specific identity elements in the cognate tRNA (34), exacerbating the difficulties for cis-acting editing sites to clear a cognate amino acid from a noncognate tRNA. This concept is demonstrated here by the fact that human AlaRS can produce Ala-tRNA^Thr^ (Figure 2A), due to its relaxed specificity toward the relocated G:U identity determinant, but is unable to correct the mistake in its own editing site (Figure 3B). Instead, the mischarged Ala-tRNA^Thr^ is edited by ThrRS, even though ThrRS itself does not create this mischarged tRNA. Thus, the function of ThrRS in this context is independent from its synthetic role but relies on its hydrolytic activity as a cross-editing factor for an error introduced by another synthetase (Figure 4). Although several free-standing trans-editing enzymes (e.g. AlaX, ProX, YbaK, DTD, ATD) have been described to clean up mischarged tRNAs that escaped from aaRSs (29,35–40), these free-standing factors often do not have clear tRNA specificity or have to rely on association with an aaRS (e.g. YbaK) to obtain tRNA specificity (35,41). In contrast, the cross-editing activity of ThrRS possesses an intrinsic specificity toward its cognate tRNA. Historically, *E. coli* PheRS was shown to be able to cross-edit Ile-tRNA^Phe^ mischarged by IleRS (42). However, the mischarging activity of IleRS toward tRNA^Phe^ appears to be extremely weak under normal conditions (15), thus the mischarging and the editing may not actually happen in cells. Nevertheless, the potential cross-editing activity of PheRS may serve as another example of how a wrong tRNA is handled *in trans* by an aaRS cognate to the mischarged tRNA species to provide intrinsic specificity.

We show here that G4:U69-containing tRNA^Thr^ are strictly conserved in vertebrates (Figure 1B), and that these mischargeable tRNA^Thr^(G4:U69) form the most prevalent and highly expressed isodecoders of tRNA^Thr^ (Figure 1C–F). However, no Thr-to-Ala mistranslation was detected in mammalian cells, presumably due to the ability of ThrRS to clear mischarged Ala-tRNA^Thr^ before its delivery to the translating ribosome. Interestingly, no mistranslation was detected even with the expression of the engineered tRNA^Thr^(G3:U70) with the G:U base pair relocated to enhance its mischarging by AlaRS (Supplementary Figure S5A). We have confirmed that the mischarged Ala-tRNA^Thr^(G3:U70) can still be efficiently deacylated by ThrRS (Supplementary Figure S5B), thus explaining this lack of mistranslation. In contrast, we successfully detected mistranslation with the amber tRNA^Thr^ (G3:U70) in the luciferase reporter. In this case, the tRNA^Thr^(G3:U70) was further engineered in the anticodon in order to decode amber stop codon for translation readthrough (Supplementary Figure S6A). Presumably, the engineered anticodon would affect the cognate tRNA recognition by ThrRS, thus reducing its editing efficiency and allowing mistranslation to occur (Supplementary Figure S6B). However, this artificial scenario is not present in natural cells.

It is interesting to note that the ability of ThrRS to clear mischarged Ala-tRNA^Thr^ appears to be evolutionarily conserved. Although the mischarged Ala-tRNA^Thr^ is unlikely to exist in prokaryotes, we show that *E. coli* ThrRS is able to deacylate human Ala-tRNA^Thr^ with similar efficiency as human ThrRS (Figure 3F). Thus, by co-opting an intrinsic activity of ThrRS, vertebrates were free to relax the specificity constraints on AlaRS and allow tRNA^Thr^ to access an extended sequence space, without compromising translation accuracy. The evolution of the exquisite functional network between AlaRS, ThrRS, and tRNA^Thr^(G4:U69) may reflect a general principle by which higher eukaryotes balance the need to preserve translational fidelity, while at the same time allowing functional expansion to support increasing complexity (Figure 4).

Considering the evolutionary conservation and the high expression level of tRNA^Thr^(G4:U69), we speculate that tRNA^Thr^(G4:U69) may have a biological function not directly related to translation. In recent years, it has become increasingly evident that there are many alternative functions of tRNA beyond translation (43,44), such as amino acid addition through tRNA for antibiotic biosynthesis and cell envelope remodeling (45–47), uncharged tRNAs regulating gene expression in response to the dynamics in amino acid availability (48–50), and formation of tRNA fragments for translation regulation and gene silencing (51–54). G4:U69 serves no obvious beneficial purpose in the canonical function of tRNA^Thr^. Yet, once established tRNA^Thr^(G4:U69) was fixed in the vertebrate lineage, suggesting strong selective pressure to retain this sequence element.

One possible function of the mischargeable tRNA^Thr^(G4:U69) may be related to stress sensing and response. It has been reported that oxidative stress induces tRNA mischarging by *Salmonella* PheRS to enhance translational quality control by increasing the rate of editing (55). Interestingly, a high-throughput sequencing method to determine the level of charged tRNA revealed that while most cytosolic tRNAs are charged at levels over 80% in HEK293T cells, tRNA^Ser^ and tRNA^Thr^ are constitutively charged at lower levels (56). The reason and purpose for lower charging levels of these two tRNA species were not clear, however, uncharged tRNA is a well-known activator of the GCN2 pathway in response to amino acid starvation and other stresses (57). The AlaRS/ThrRS-mediated mischarging and deacylation cycle described here may contribute to the relatively low charging levels of tRNA^Thr^ and may serve as a sensitized system for stress sensing (Figure 4).

**Figure 5.**
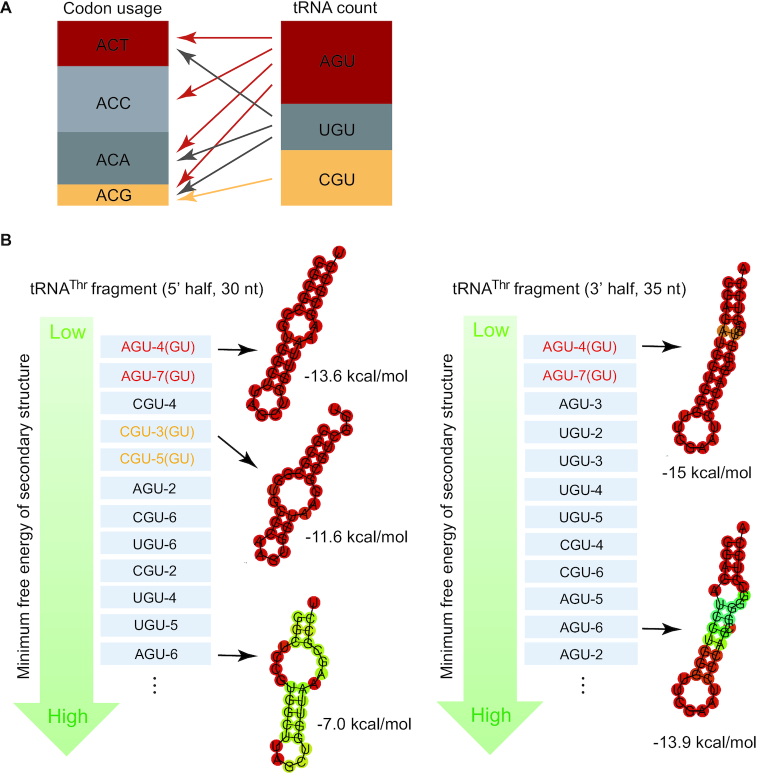
Lack of correlation between Thr codon usage and tRNA^Thr^ abundance and potential fragmentation of tRNA^Thr^(G4:U69). (**A**) Comparison of the estimated relative frequency of the Thr codon usage and the relative counts of different tRNA^Thr^ isoacceptor families in the human genome indicates a lack of correlation between codon usage and tRNA abundance. (**B**) G4:U69 base pair may play a role in stabilizing tRNA^Thr^ fragments. The fragments (5′- and 3′-halves) are listed according to their predicted minimum free energy from low to high, putting the most stable tRNA fragments on the top, which are from G4:U69-containing tRNA^Thr^. The secondary structure of potential tRNA^Thr^ fragments and their minimum free energy are predicted by RNAfold server. The secondary structure of representative fragments is shown aside. The sizes of tRNA 5′- and 3′-halves are approximated based on the size ranges of tRNA fragments reported so far.

Additionally or alternatively, tRNA^Thr^(G4:U69) may serve regulatory functions through its fragmentation (Figure 4). Increasing evidence suggests that tRNA halves or tRNA-derived fragments (tRFs) could regulate translation and gene expression. From our northern blot analysis, we could clearly detect the differential occurrence of tRFs (or tRNA halves) derived from tRNA^Thr^(G4:U69) in tissues (Figure 1E). Especially in small intestine, lung, and pancreas, the level of tRNA^Thr^(G4:U69)-CGU fragments is comparable to the full length tRNA^Thr^(G4:U69)-CGU. Interestingly, codon usage corresponding to tRNA^Thr^-CGU is much lower than other threonine codons (Figure 5A). Based on the pairing rules, tRNA^Thr^-CGU can only decode ACG codon, which has a low usage, while other isoacceptors with AGU and UGU anticodons can be used for decoding all or almost all four codons and can compensate for the absence of tRNA^Thr^-CGU (Figure 5A). Therefore, the necessity of tRNA^Thr^-CGU for translation is relatively low, suggesting tRNA^Thr^-CGU, especially G4:U69-containing tRNA^Thr^-CGU (see below), may be required for alternative functions beyond translation.

It was reported that a stable stem-loop secondary structure is crucial for tRNA halves to avoid degradation by RNases (58). Prediction of the secondary structure of tRNA^Thr^-derived tRNA halves by the RNAfold server shows that both 5′- and 3′-halves of tRNA^Thr^(G4:U69)-AGU and the 5′ half of tRNA^Thr^ (G4:U69)-CGU form stable stem-loop structures (Figure 5B). In fact, tRNA^Thr^(G4:U69)-AGU halves rank on the top in stability among all tRNA^Thr^ halves. Interestingly, although there is only one other nucleotide difference between the 5′ half of tRNA^Thr^(G4:U69)-AGU4 and that of tRNA^Thr^(U4:G69)-AGU6 (in addition to the G4/U4 position), the former exhibits a much more stable secondary structure than the latter (Figure 5B). Moreover, prediction by RNAstructure bifold showed a high probability for homo- or heterodimer formation by tRNA^Thr^(G4:U69)-AGU 5′ halves (Supplementary Figure S9A), which can further protect the tRNA fragments from degradation (58). In addition, based on the database of tRNA fragments (Mintbase) in human tissues, fragments of tRNA^Thr^(G4:U69), especially tRNA^Thr^(G4:U69)-CGU, are the most abundant populations among all sequences derived from tRNA^Thr^ (Supplementary Figure S9B). Therefore, the G4:U69 base pair may play a role in stabilizing tRNA^Thr^ fragments.

Finally, mischarging tRNA^Thr^ with alanine may in itself serve as a signal for production of tRFs (Figure 4). Honda *et al.* reported a novel type of tRNA-derived small RNA, named hormone-dependent tRNA-derived RNAs (SHOT-RNAs), which enhance cell proliferation in breast and prostate cancers. Unlike other tRFs, SHOT-RNAs are exclusively produced from charged tRNAs, where the 3′ amino acid may play a role in tRNA selectivity by ANG (59). In light of our finding, it is possible that mischarging of tRNA^Thr^(G4:U69) with Ala may be important for fragmentation of tRNA^Thr^(G4:U69), as mischarged tRNA^Thr^ may have a lower binding affinity to EF1A and therefore is more accessible to be captured and cleaved by RNases.

In summary, the properties of tRNA^Thr^(G4:U69) discussed above suggest that it serves a function that would explain its vertebrate-specific conservation. Further studies, such as vertebrate-related phenotype characterization under manipulation of tRNA^Thr^(G4:U69), are required to elucidate the roles of these specific tRNAs. Importantly, the fact that these specific, mischargeable tRNAs can exist in evolution is likely due to the efficient and intrinsic cross-editing function of ThrRS demonstrated in this study.

## Supplementary Material

gkaa469_Supplemental_Files
