# Supplementary material for: Cross-editing by a tRNA synthetase allows vertebrates to abundantly express mischargeable tRNA without causing mistranslation
Source: Nucleic Acids Res. 2020 Jun 2;48(12):6445–57. doi: 10.1093/nar/gkaa469 (PMC7337962; doi:10.1093/nar/gkaa469)
Supplement: gkaa469_Supplemental_Files [file gkaa469_supplemental_files.zip › Supplementary Figures 2020.05.19.pdf]

Supplementary Figures

Chen et al.

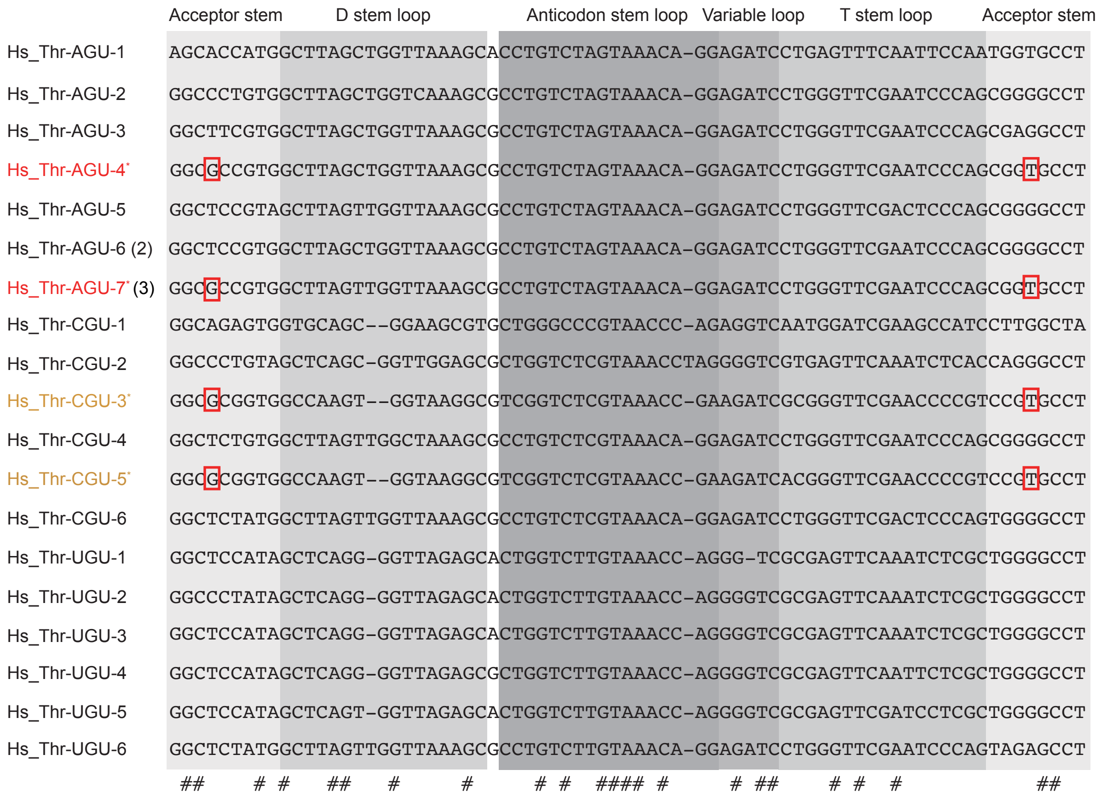

Figure S1. (Related to Figure 1) Sequence alignment of human $tRNA^{Thr}$. The G:U base pair is marked with red frame. The identical nucleotides among tRNAThr are indicated by "#".

A

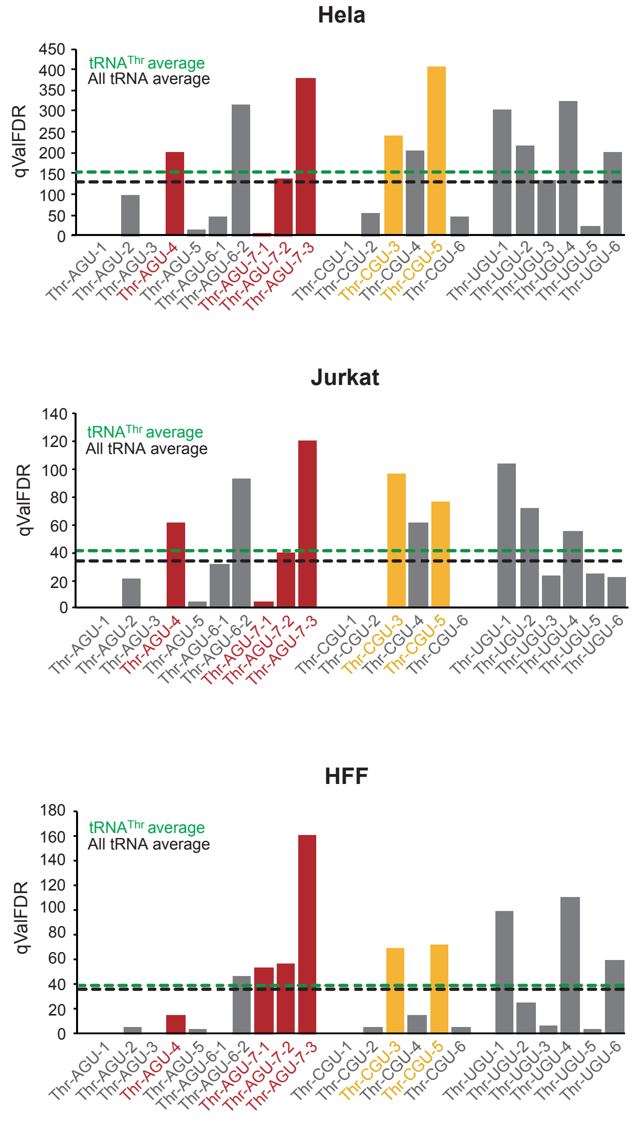
Hela
qValFDR
tRNA^{Thr} average
All tRNA average
450
400
350
300
250
200
150
100
50
0
Thr-AGU-1
Thr-AGU-2
Thr-AGU-3
Thr-AGU-4
Thr-AGU-5
Thr-AGU-6-1
Thr-AGU-6-2
Thr-AGU-7-1
Thr-AGU-7-2
Thr-AGU-7-3
Thr-CGU-1
Thr-CGU-2
Thr-CGU-3
Thr-CGU-4
Thr-CGU-5
Thr-CGU-6
Thr-UGU-1
Thr-UGU-2
Thr-UGU-3
Thr-UGU-4
Thr-UGU-5
Thr-UGU-6
Jurkat
qValFDR
tRNA^{Thr} average
All tRNA average
140
120
100
80
60
40
20
0
HFF
qValFDR
tRNA^{Thr} average
All tRNA average
180
160
140
120
100
80
60
40
20
0

B

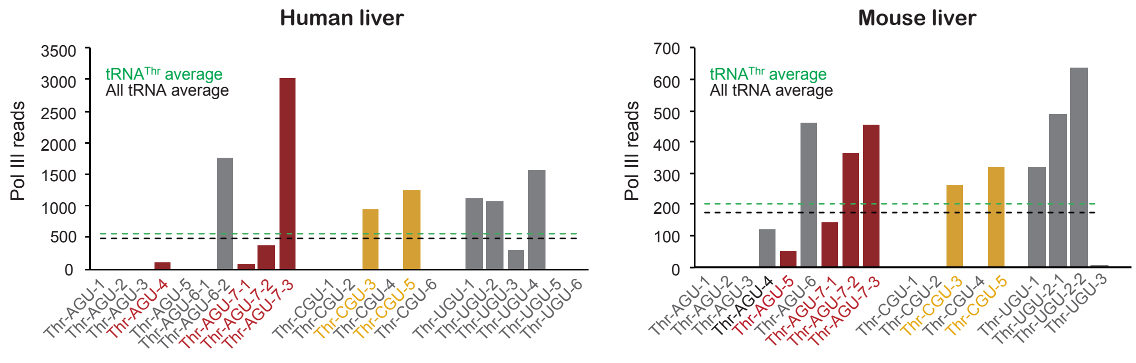
Human liver
Pol III reads
tRNA^{Thr} average
All tRNA average
3500
3000
2500
2000
1500
1000
500
0
Thr-AGU-1
Thr-AGU-2
Thr-AGU-3
Thr-AGU-4
Thr-AGU-5
Thr-AGU-6-1
Thr-AGU-6-2
Thr-AGU-7-1
Thr-AGU-7-2
Thr-AGU-7-3
Thr-CGU-1
Thr-CGU-2
Thr-CGU-3
Thr-CGU-4
Thr-CGU-5
Thr-CGU-6
Thr-UGU-1
Thr-UGU-2
Thr-UGU-3
Thr-UGU-4
Thr-UGU-5
Thr-UGU-6
Mouse liver
Pol III reads
tRNA^{Thr} average
All tRNA average
700
600
500
400
300
200
100
0
Thr-AGU-1
Thr-AGU-2
Thr-AGU-3
Thr-AGU-4
Thr-AGU-5
Thr-AGU-6
Thr-AGU-7-1
Thr-AGU-7-2
Thr-AGU-7-3
Thr-CGU-1
Thr-CGU-2
Thr-CGU-3
Thr-CGU-4
Thr-CGU-5
Thr-UGU-1
Thr-UGU-2-1
Thr-UGU-2-2
Thr-UGU-3

C

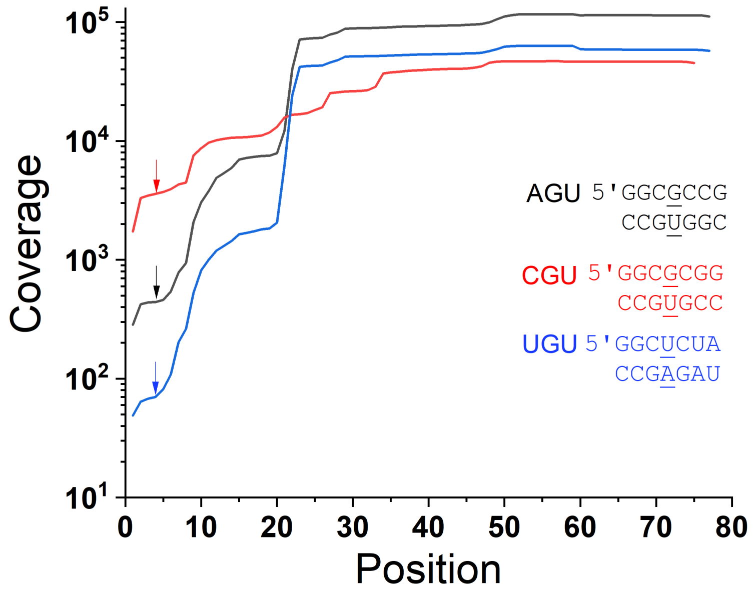

Figure S2. (Related to Figure 1) Pol III ChIP-Seq analysis. (A) ChIP-Seq analysis showing Pol III binding to $tRNA^{Thr}$ genes in HeLa, Jurkat, and HFF cells. (B) ChIP-Seq analysis showing Pol III binding to $tRNA^{Thr}$ genes in human and mouse liver tissues. The $tRNA^{Thr}$ genes containing a G4:U69 base pair are marked in red and yellow, corresponding to AGU and CGU isoacceptors, respectively. The black and green dash lines indicate the average Pol III binding level among all tRNA genes and all $tRNA^{Thr}$ genes, respectively. (C) DM-tRNA-seq coverage of HEK293T tRNAThr. Isodecoders in each acceptor family shown correspond to Thr-AGU-7; Thr-CGU-5; Thr-UGU-6 in Fig. 1D. The acceptor stem sequences for these specific isodecoders are also shown with position 4 and 69 underlined. Reads that reach position 4 are from full-length tRNAs and indicated by arrows. All reads start with the 3' CCA in this experiment because of the design of DM-tRNA-seq procedure. The drop-off along the tRNA is mostly due to the RT stops caused by several modifications and/or tRNA structure.

A

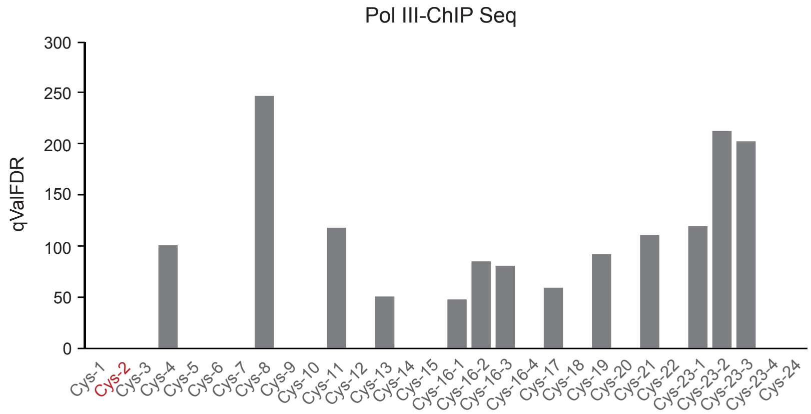

B

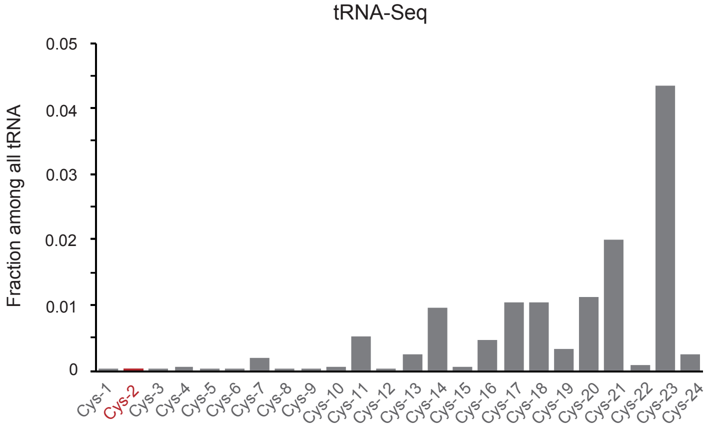

Figure S3. (Related to Figure 1) Pol III ChIP-Seq and tRNA-Seq analysis of $tRNA^{Cys}$ genes in HEK293 cells. **(A)** ChIP-Seq analysis showing Pol III binding to $tRNA^{Cys}$ genes in HEK293 cells. **(B)** The level of $tRNA^{Cys}$ isoacceptors in HEK293T cells as estimated by tRNA-seq analysis. The single $tRNA^{Cys}$ isoacceptor containing a G4:U69 base pair is highlighted in red.

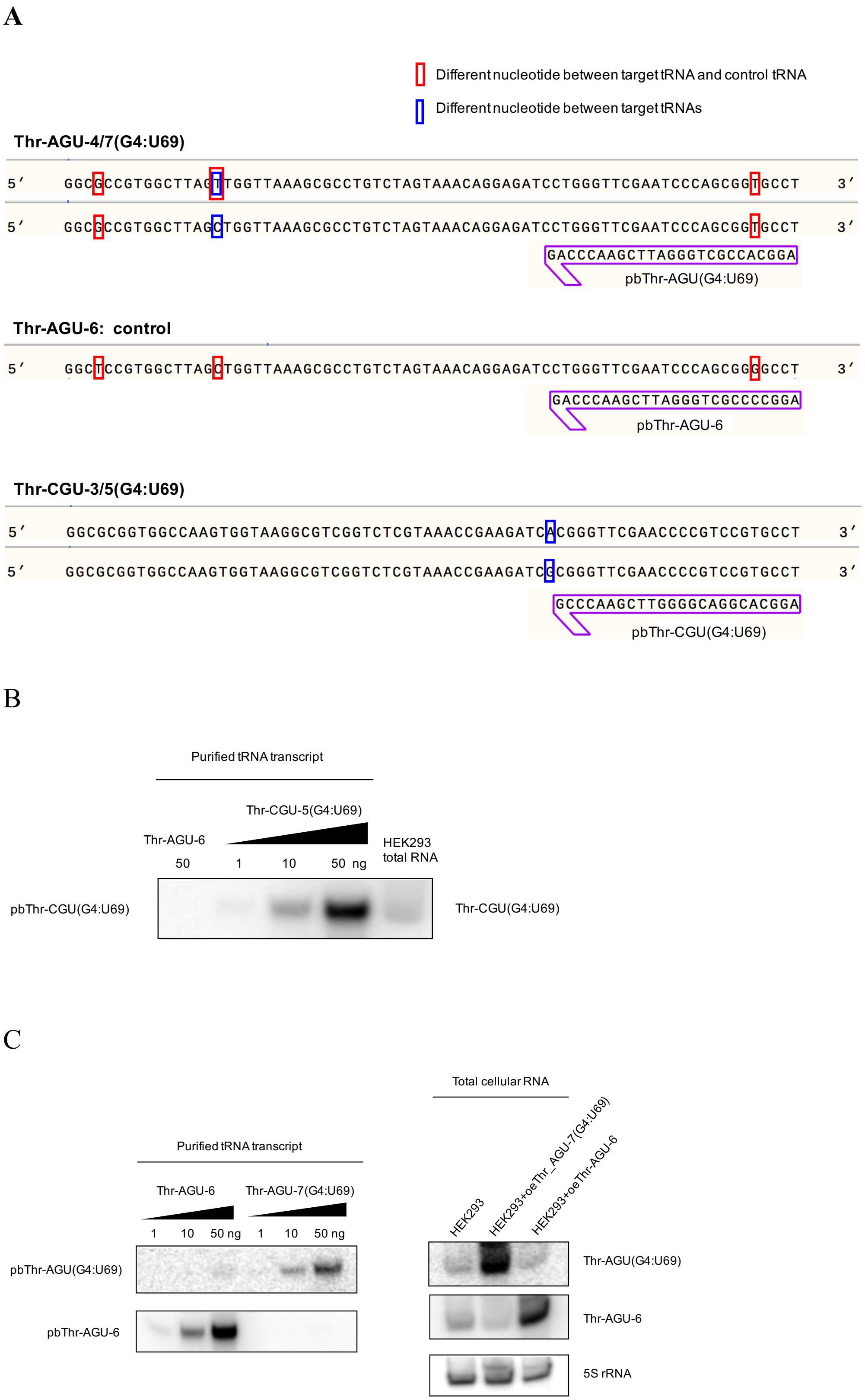
A
Different nucleotide between target tRNA and control tRNA
Different nucleotide between target tRNAs
Thr-AGU-4/7(G4:U69)
5′ GGCGCCGTGGCTTAGTTGGTTAAAGCGCCTGTCTAGTAAACAGGAGATCCTGGGTTCGAATCCCAGCGGTGCCT 3′
5′ GGCGCCGTGGCTTAGCTGGTTAAAGCGCCTGTCTAGTAAACAGGAGATCCTGGGTTCGAATCCCAGCGGTGCCT 3′
GACCCAAGCTTAGGGTCGCCACGGA
pbThr-AGU(G4:U69)
Thr-AGU-6: control
5′ GGCTCCGTGGCTTAGCTGGTTAAAGCGCCTGTCTAGTAAACAGGAGATCCTGGGTTCGAATCCCAGCGGGGCCT 3′
GACCCAAGCTTAGGGTCGCCCCGGA
pbThr-AGU-6
Thr-CGU-3/5(G4:U69)
5′ GGCGCGGTGGCCAAGTGGTAAGGCGTCGGTCTCGTAAACCGAAGATCACGGGTTCGAACCCCGTCCGTGCCT 3′
5′ GGCGCGGTGGCCAAGTGGTAAGGCGTCGGTCTCGTAAACCGAAGATCGCGGGTTCGAACCCCGTCCGTGCCT 3′
GCCCAAGCTTGGGGCAGGCACGGA
pbThr-CGU(G4:U69)
B
Purified tRNA transcript
Thr-CGU-5(G4:U69)
Thr-AGU-6
HEK293 total RNA
50
1
10
50 ng
pbThr-CGU(G4:U69)
Thr-CGU(G4:U69)
C
Total cellular RNA
Purified tRNA transcript
Thr-AGU-6
Thr-AGU-7(G4:U69)
1
10
50 ng
1
10
50 ng
HEK293
HEK293+oeThr_AGU-7(G4:U69)
HEK293+oeThr-AGU-6
pbThr-AGU(G4:U69)
pbThr-AGU-6
Thr-AGU(G4:U69)
Thr-AGU-6
5S rRNA

D

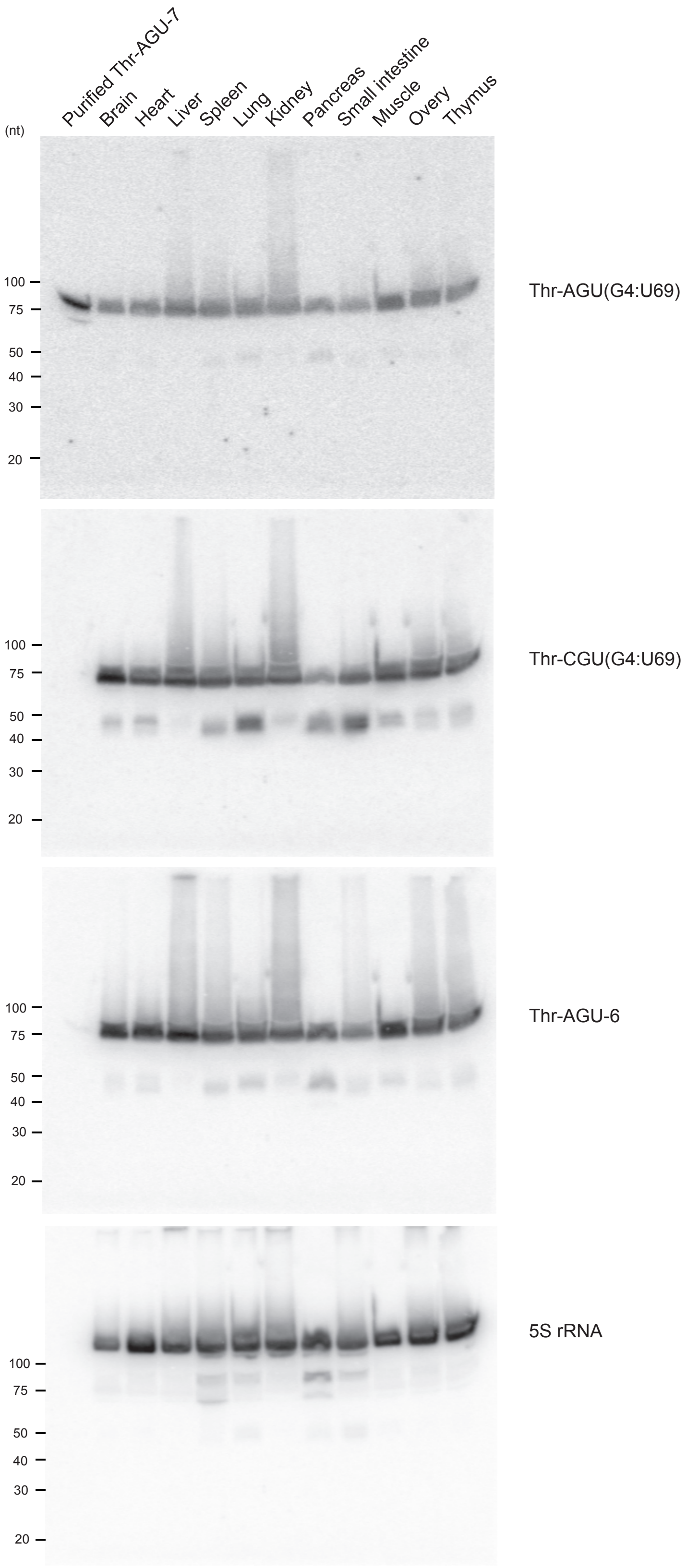
Purified Thr-AGU-7
Brain
Heart
Liver
Spleen
Lung
Kidney
Pancreas
Small intestine
Muscle
Overy
Thymus
(nt)
100
75
50
40
30
20
Thr-AGU(G4:U69)
Thr-CGU(G4:U69)
Thr-AGU-6
5S rRNA

Figure S4. (Related to Figure 1) Specific detection of tRNA^{Thr}(G4:U69)-AGU and tRNA^{Thr}(G4:U69)-CGU isoacceptors by Northern blot analysis. **(A)** Sequence comparison of tRNA^{Thr} genes and the probe design. **(B, C)** Examination of probe specificity with purified tRNA transcripts and detection of endogenously or overexpressed tRNA in HEK293 cells. A non-G4:U69 containing Thr-AGU-6 was used as the control for specificity. Thr-AGU-6 has the highest sequence similarity to all 4 tRNA^{Thr}(G4:U69)-AGU and tRNA^{Thr}(G4:U69)-CGU isoacceptors, and therefore serves as the most stringent control for probe specificity. 5S rRNA was used as a loading control. **(D)** Full blots of Figure 1E.

A

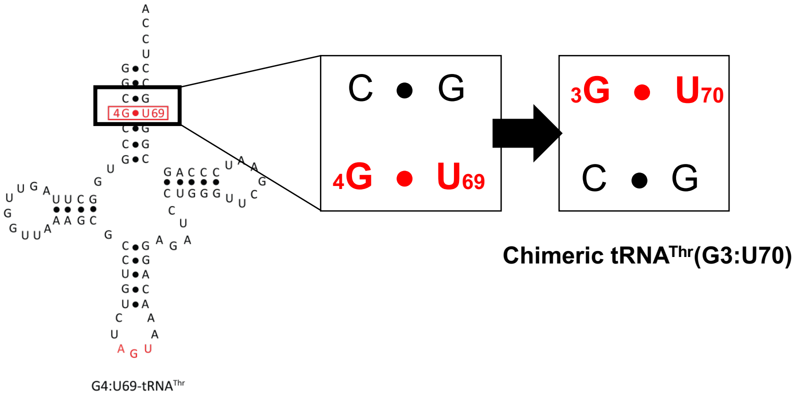

B

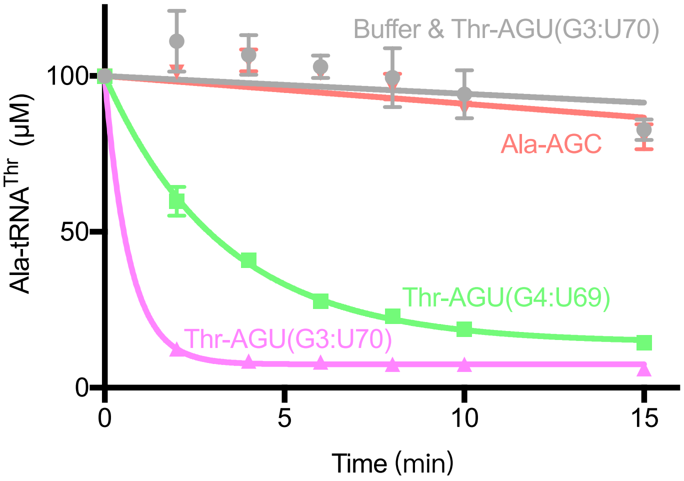

Figure S5. (Related to Figure 2) **(A)** Sequence and secondary structure of the chimeric tRNA^{Thr}(G3:U70). **(B)** Editing of Ala-tRNA^{Thr}(G3:U70) by ThrRS.

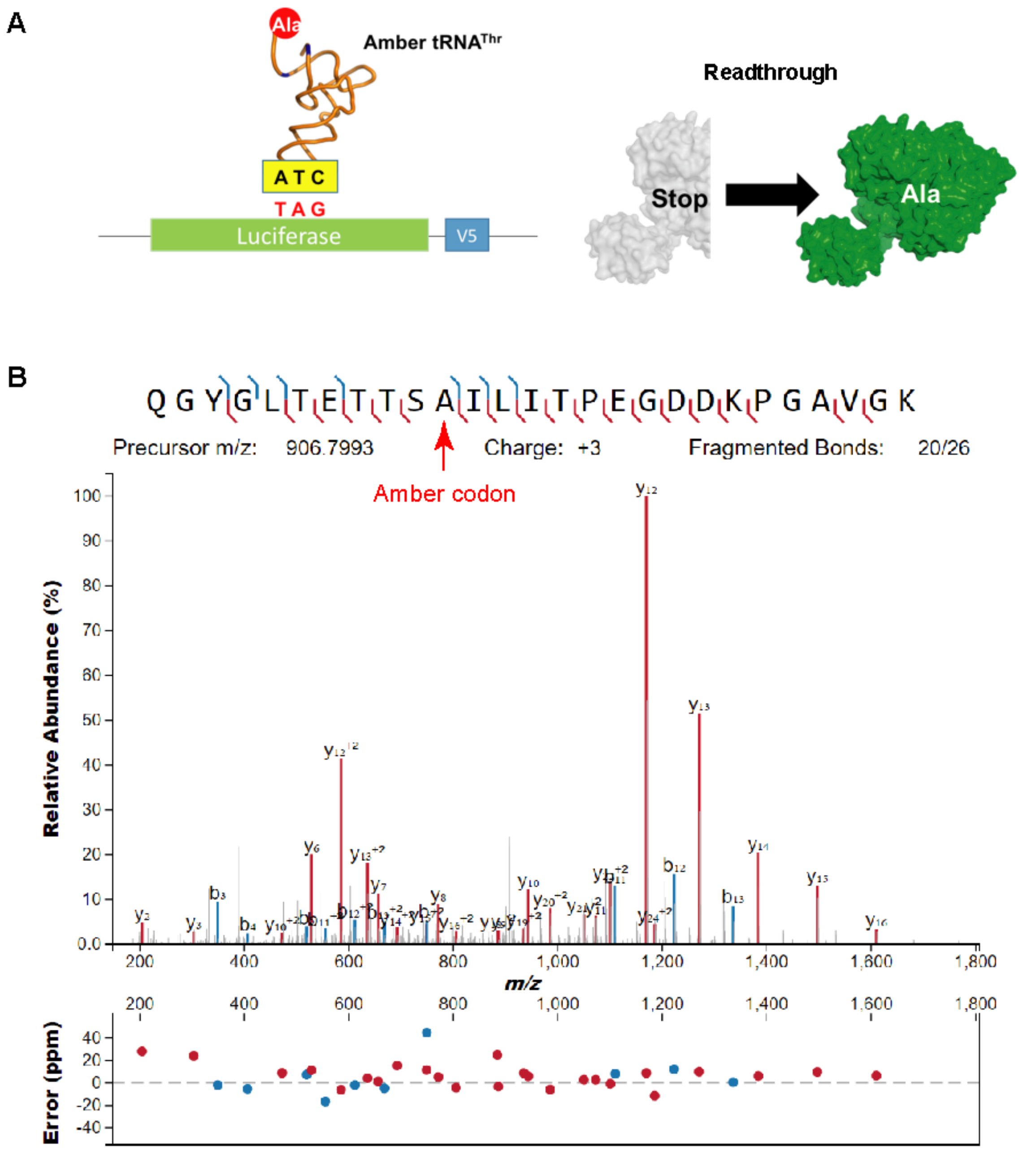

Figure S6. (Related to Figure 2) Strategy to detect alanine misincorporation through mischarging tRNA^{Thr}. **(A)** Illustration of the readthrough strategy in capturing report protein for detecting potential alanine misincorporation. **(B)** Representative MS/MS spectra demonstrating alanine is incorporated at the amber codon site only when the chimeric tRNA^{Thr}(G3:U70) is expressed.

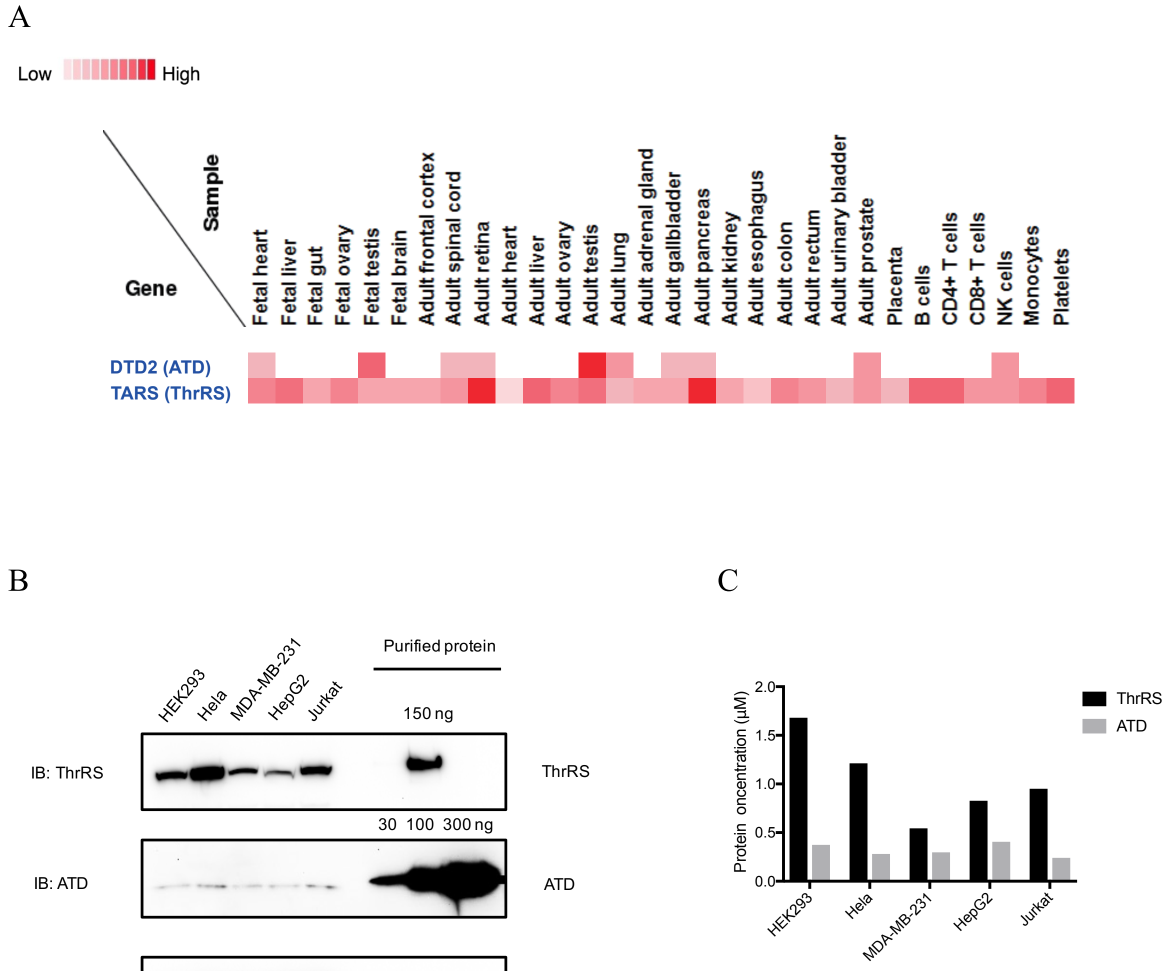

Figure S7. (Related to Figure 3) **(A)** Protein expression analysis of ATD (also known as DTD2) and ThrRS in various human tissues and cell types based on the HUMAN PROTEOME MAP database (http://www.humanproteomemap.org/). **(B, C)** Expression and quantification analysis of ThrRS and ATD in different human cell lines by Western blot analysis. Purified proteins of ThrRS and ATD were used for quantification purpose.

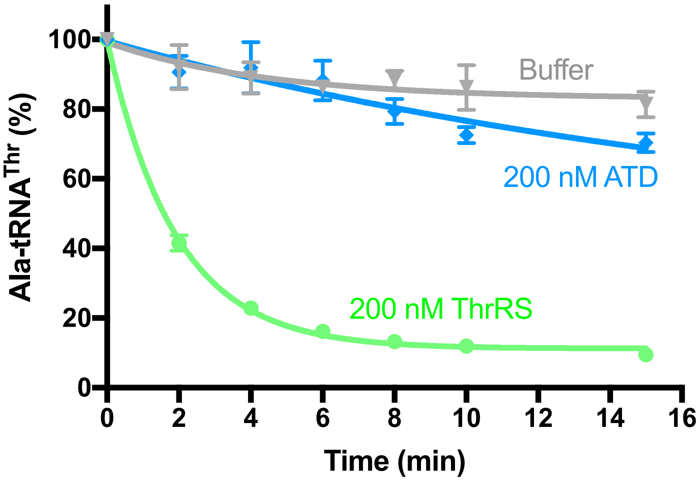

Figure S8. (Related to Figure 3) Comparison of human ThrRS and ATD in editing Ala-$tRNA^{Thr}$.

A

Homodimer of 5'-half of Thr-AGU-4(GU)　　　Heterodimer of 5'-half of AGU-4(GU) and AGU-7(GU)

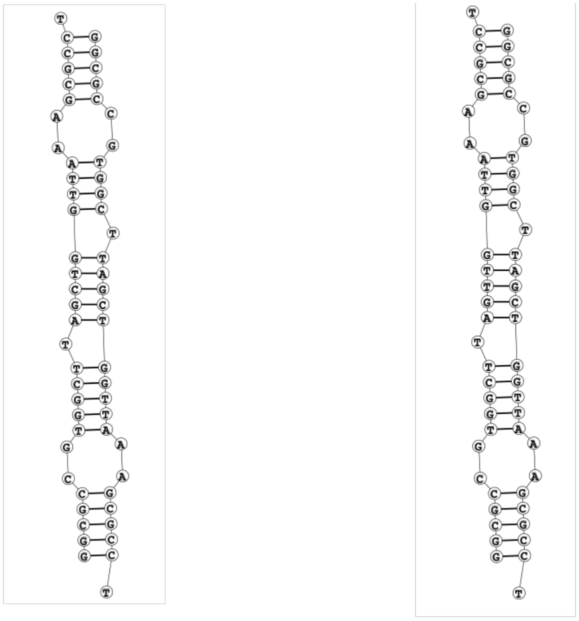

-35.5 kcal/mol　　　-33.1 kcal/mol

B

No.	tRNA	Type	Fragment length (nt)	No. of dataset	Maximum RPM
1	Thr-CGU (GU)	5' tRF	24	9105	1408
2	Thr-CGU (GU)	5' tRF	23	4469	846
3	Thr-AGU (GU)	3' tRF	16	6747	405
4	Thr-UGU	3' tRF	17	7304	330

Figure S9. (Related to Figure 5) $tRNA^{Thr}$ fragmentation. **(A)** Stable homo- and hetero-dimer formation of tRNA fragments derived from G4:U69-containing $tRNA^{Thr}$(AGU) as predicted by RNAstructure server. The 5'-halves of 30 bp were used in the prediction. **(B)** Annotated $tRNA^{Thr}$ fragments in MINTbase (https://cm.jefferson.edu/MINTbase/) indicating top 3 most abundant

tRNA^{Thr} fragments are from G4:U69-containing tRNA^{Thr}. RPM of 5'-halves of Thr-CGU(GU) includes the signal from both Thr-CGU-3 and Thr-CGU-5, because they share the same sequence at the 5'-half. Similarly, RPM of 3'-halves of Thr-AGU(GU) includes all four Thr-AGU(GU) (i.e., Thr-AGU-4, Thr-AGU-7-1, Thr-AGU-7-2, and Thr-AGU-7-3) as they share the same sequence at the 3'-half.
